# Supplementary material for: JmjC domain-containing histone demethylase gene family in Chinese cabbage: Genome-wide identification and expressional profiling
Source: PLoS One. 2024 Nov 15;19(11):e0312798. doi: 10.1371/journal.pone.0312798 (PMC11567544; doi:10.1371/journal.pone.0312798)
Supplement: S1 Table — It includes the gene name, gene ID, forward primer sequence, reverse primer sequence, and the gene name and gene ID of the closest Arabidopsis thaliana homolog for each of the BrJMJ genes. (PDF) [file pone.0312798.s001.pdf]

S1 Table. Detailed information on *BrJMJs* in Chinese cabbage. It includes the gene name, gene ID, forward primer sequence, reverse primer sequence, and the gene name and gene ID of the closest *Arabidopsis thaliana* homolog for each of the *BrJMJ* genes.

Gene name	Gene ID	Forward primer (5'—3')	Reverse primer (5'—3')	Gene name of the closest *Arabidopsis thaliana* homolog	Gene ID of the closest *Arabidopsis thaliana* homolog
BrJMJ1	BraA01g011450.3C	GGAAGCCATGC TGCGTCCTT	CTGCGCTGGAC AGCTCGATA	*AtJMJ*14	AT4G20400
BrJMJ2	BraA01g012140.3C	AGTTTCGCTGCA ACCGCTCC	CTAGCGAGCTT TGTCGACGC	*AtJMJ*27	AT4G00990
BrJMJ3	BraA02g033320.3C	CCCTTCGACTTG CTGAGTGGG	TCTCCAGGAAT GAGTCGGGCA	*AtJMJ*13	AT5G46910
BrJMJ4	BraA03g009260.3C	AATATGCCGAC GGGATGCGG	AGAACAGGCC TCCCAGTCAC	*AtJMJ*30	AT3G20810
BrJMJ5	BraA03g020590.3C	CTGGTGGGGAC AAAGGCAAGA	TTAGGGTCCAC AGGCCCTGAC	*AtJMJ*19	AT2G38950
BrJMJ6	BraA03g029710.3C	AGAGCGCCTTC CAAGGCATA	ACGCAATGTAC GTCTTGGGTCC	*AtJMJ*26	AT1G11950
BrJMJ7	BraA03g029730.3C	CTGTTGATCTGG CTGAAGGCGA	ATTTCCCTGCA AGCCCTCCAC	*AtJMJ*26	AT1G11950
BrJMJ8	BraA03g049530.3C	AACGCTTATTCT GTTTCTTCAGAC G	AGCAACGTGCT CAGGTGAGAG	*AtJMJ*27	AT4G00990
BrJMJ9	BraA04g024870.3C	TCCTGGAACCC ATGCAACGTC	ACCGCACGATA AACCGGGAC	*AtJMJ*15	AT2G34880
BrJMJ10	BraA04g025520.3C	TGCGGGAAGAA CGGGACTCT	ACGCACTCCCT CTCGCAGTT	*AtJMJ*15	AT2G34880
BrJMJ11	BraA05g006830.3C	CGCGGTCCCAG GAAAACTCT	ACATCTGCAAC GGTTGGCTCT	*AtJMJ*19	AT2G38950
BrJMJ12	BraA05g026320.3C	ATACTTGCTCAG GTTGTTGGCA	AGCTCCACCAC CTTTGGAAACT	*AtJMJ*29	AT1G62310
BrJMJ13	BraA05g038070.3C	TTTGCGCAAGA AATGGGCCG	TGTGCAGCATG CTTCCTCTTCA	*AtJMJ*24	AT1G09060
BrJMJ14	BraA06g005740.3C	GGGCGTACCAC GCTGGATTC	GCAGCCCCAA GCAGCAATTT	*AtJMJ*16	AT1G08620
BrJMJ15	BraA06g018530.3C	GCTTGGCATGT GGAGGACCAT	CCTGCAACATG GTATCCCAGCT C	*AtJMJ*12	AT3G48430
BrJMJ16	BraA06g020720.3C	TGGGTTCCGAA ATCGACCGC	CTCCGGAGAG GTAAGCGGGA	*AtJMJ*31	AT5G19840

BrJMJ17	BraA07g041420.3C	GGTTGGTGGCACTGCATCCT	AGAAGCCCAGCACGACACAC	*AtJMJ*21	AT1G78280
BrJMJ18	BraA08g010460.3C	CACGGATTCAGACCGTGGACC	TCTGCCTCGGGAGAAGAGCC	*AtJMJ*18	AT1G30810
BrJMJ19	BraA08g031280.3C	AGCCAACTGCGAGGTGAAGAT	ACTCATCGCAGTGACGTGGC	*AtJMJ*25	AT3G07610
BrJMJ20	BraA08g032870.3C	ACGAGCAAACTCGATCCGGC	GTGCTTCTCGCACACGGTCT	*AtJMJ*32	AT3G45880
BrJMJ21	BraA08g033080.3C	TGCAGCCTTCTGGGAGTCCT	AACCTGTGGCCGCGAGTAAG	*AtJMJ*16	AT1G08620
BrJMJ22	BraA09g000040.3C	CCCTGCCCTCCAAAGGAACG	AGCAGTATCCACATCCGGTGG	*AtJMJ*26	AT1G11950
BrJMJ23	BraA09g007640.3C	AGTCCAGAGTTCAGGAGTATCCAAA	ACAAAGCGATAATCAGAGCAGCTT	*AtJMJ*20	AT5G63080
BrJMJ24	BraA09g013190.3C	TGCTTCAGGCCATTCGGCAT	GTTGACGGCCTCTGCACAGTTT	*AtJMJ*17	AT1G63490
BrJMJ25	BraA09g016000.3C	TGTCCCAACGCCCTGAAACG	ACCAGCATTCCACTTTGCGGA	*AtJMJ*28	AT4G21430
BrJMJ26	BraA09g022420.3C	CGGGACAACGCGAACTCTGT	CAGCTGCAAGTTTGAGTGGCG	*AtJMJ*13	AT5G46910
BrJMJ27	BraA09g034190.3C	GCCGAGGAGAAGTTCGGCTT	TCCTCCACAGACGGTGTCCAT	*AtJMJ*18	AT1G30810
BrJMJ28	BraA10g029240.3C	AGCCAAGCACGGAGTCCAAC	TAAAACGCCGAGATGGGCCG	*AtJMJ*22	AT5G06550
BrJMJ29	BraA10g032100.3C	GATCGCACGTTCGCCTGGC	CCCTACGCACAGCAGCTTCCTT	*AtJMJ*11	AT5G04240
BrActin7	BraA03g004000.3C	CCGAGCGAGAAATCGTCCGT	ATTCCGATGAGCGACGGCTG		
