# Supplementary material for: JmjC domain-containing histone demethylase gene family in Chinese cabbage: Genome-wide identification and expressional profiling
Source: PLoS One. 2024 Nov 15;19(11):e0312798. doi: 10.1371/journal.pone.0312798 (PMC11567544; doi:10.1371/journal.pone.0312798)
Supplement: Attachment — Submitted filename: Response letter-7.30.docx [file pone.0312798.s002.docx]

Dear Steven G. Gray Editor:

Thank you for your letter and for the your comments concerning our manuscript entitled “JmjC Domain-containing Histone Demethylase Gene Family in Chinese Cabbage: Genome-wide Identification and Expressional Profiling”. Those comments are all valuable and very helpful for revising and improving our paper, as well as the important guiding significance to our researches. We have studied comments carefully and have made correction which we hope meet with approval. The changes are highlighted in red. The main corrections in the paper and the responds to your comments are as flowing:

**Point 1 (Reviewer1):** The manuscript by Fengrui and colleagues presents a phylogenetic analysis of the Jumonji domain protein family in *Brassica rapa* and a brief gene expression analysis by RT-PCR. Despite its potential interest for the Plant Science community, the article is not sound.
The phylogenetic analysis is quite simple because they only downloaded sequences from a B. rapa genome version 3. It is unclear why they did not use a more recent version of the genome. They then constructed a phylogenetic tree following standard procedures and using MEGA software. Additionally, it is quite difficult to follow their work because they name the B. rapa proteins as BrJMJX but do not provide the corresponding *B. rapa* gene numbers or *Arabidopsis* paralog gene numbers.
In fact, another problematic issue with the manuscript is the lack of proper introduction. They miss and do not mention the vast literature on JmjC proteins in *Arabidopsis*, rice, and other plants. They also do not cite any plant-specific review on *JmjC* functions (doi:10.3389/fpls.2021. 687416; doi:10.1016/j.isci.2020.101715) or recent key phylogenetic analysis of plant JmjC porteins (doi:10.1104/pp. 15.00520). Even more concerning, they do not mention recent high-profile B. rapa publications on JmjC genes (doi:10.1038/s41467-024-49721-z; doi: 10.1111/pce.1425). Given all these issues, I am very skeptical about the quality and reliability of the results presented.

Response to comment: Thank you very much for your professional advice. As stated by the reviewer.

(1) Reasons for using ‘*Brara_Chiifu_V3.0*’ reference genome: This version is widely used and has been widely used in the last two years of articles (DOI:10.3390/agronomy12122976; DOI:10.3390/genes13050761; DOI:10.1186/s12864023097739;DOI:10.3390/plants13081118;DOI:10.1007/s10722024019254). And the genome data of this version is relatively complete and accurate, which helps us to identify and analyze the whole genome.
(2) According to the reviewer's comments, B. rapa gene numbers are reflected in Table S1 (Line 205). In the introduction part of the manuscript, the research progress of JmjC gene was mentioned, and the discovery of JmjC gene family in *Arabidopsis*, *Oryza sativa*, *Rosa Chinensis*, *Zea mays* L and *Gossypium hirsutum* (Line 70-72, 79, 84, 90-94) was cited. In the discussion section, the results of our study are compared with those of the JmjC gene family in other species (*Arabidopsis*, *Betula pendula*, *Glycine max*, *Pyrus bretchneideri*, *Fragaria ananassa*, *Vitis vinifera* and so on) (Line 388-392, 427-428, 476-482 and so on). Plant-specific comments on JmjC gene function were added to the manuscript results (Line 351) and discussion (Line 432-433). Recent high-profile B. rapa publications on *JmjC* genes are added to the manuscript introduction (Line 102) and discussion (Line 464-477).
(3) In the results section, the results of the phylogenetic analysis of our manuscript were further improved according to the phylogenetic tree analysis of reference (DOI:10.1104/pp.15.00520). The analysis and discussion of the branching of evolutionary tree are also added (Line 210-213, 217-218, 223-227). In the discussion section, the results of recent key phylogenetic analyses of plant JmjC porteins are compared with those of our evolutionary tree (Line 396-406).

**Point 2 (Reviewer2):** This manuscript describes the genome-wide characterization and expression analysis of *JmjC* genes in Chinese cabbage. For the manuscript improvement, there are some points required more consideration and revision.
(1) Introduction can be improved, the information is too lengthy and jumbled on this topic, the important recent results should be summarized in concise sentences.

Response to comment: Thank you very much for your professional advice. According to the reviewer's comments, we have deleted the repetitive and useless information in the introduction of the manuscript (Line 74, 79, 81, 88, 90, 93, 102). It also condensed recent research findings, making the introduction simple and clear (Line 84-86, 93, 95-97).
(2) Please offer more information about the experimental material " Chinese cabbage variety “Xiayangzao 50”", including its accession number. Is it an inbred line?
Response to comment: Thank you very much for your professional advice. The specific information of the Chinese cabbage cultivar ‘Xiayang Early 50' is supplemented in the Plant materials section of the Materials and Methods section (Line 117-118).
(3) The phylogenetic tree lacks bootstrap support for branching.
Response to comment: Thank you very much for your professional advice. In the results section, the results of the phylogenetic analysis of our manuscript were further improved according to the phylogenetic tree analysis of reference (DOI:10.1104/pp.15.00520). The analysis and discussion of the branching of evolutionary tree are also added (Line 210-213, 217-218, 223-227).
(4) The figures in manuscript do not have sufficient resolution/clarity, and out of order.
Response to comment: Thank you very much for your professional advice. The clarity of the figures in the manuscript was adjusted by using Photoshop software. And in the part of the manuscript Figures, the figures with weak clarity is replaced. The order of the figures was re-adjusted (Line 765-806).
(5) Provide detailed description of osmotic, salt, cold, and heat stress treatment process in Materials and Method.
Response to comment: Thank you very much for your professional advice. According to the reviewer's suggestion, in the Materials and Method, we describe in detail the process of osmotic, salt, cold and heat stress treatment (Line 126-128). Specific content: Among them, osmotic stress (20% PEG6000) and salt (200 mM NaCl) were hydroponic in the seedling room. Hydroponic was performed in the incubator under cold (4.0 ℃) and heat (38 ℃) stress.
(6) The protein structural domains, PHD, DM, AT-hook, the firstly introduced abbreviation should be given the full name and provide references.
Response to comment: Thank you very much for your professional advice. In the protein structural domains analysis in the results section, we gave the full names and provided references for the leading abbreviations of JmjC (Jumonji C), JmjN (Jumonji N), DEXDc (DEAD-like helicases superfamily), HELICc (Helicase superfamily c-terminal domain), FYRN (“FY-rich” domain N-terminal), FYRC (“FY-rich” domain C-terminal), ARID (AT-rich interaction domain), PHD (Plant Homeodomain), ZnF_C2H2 (zinc-finger of C2H2-type) domain, F-box (FBOX a receptor for ubiquitination targets), DM (Dsx and Mab-3) and AT-hook (DNA binding domain with preference for A/T rich regions) and RING (really interesting new gene) (Line 241-244, 246-247, 251-257). The full names of the firstly introduced abbreviation are described in Figure legend of 2A (Line 770-776).
(7) Fig 6B is lack of scale bar. The cd plants showed reduced leaf size. This phenotype should be examined and explained.
Response to comment: Thank you very much for your professional advice. Add the scale in Figure 6B in the lower right corner (Line 790). And Scale bar, 4 cm (Line 793). The explanation of the phenotype of the reduced leaf size of Cd plants was added in the *BrJMJ* expression under Cd stress section of the Results (Line 336-337).
(8) Figure legend of 6C is absent.

Response to comment: Thank you very much for your professional advice. Figure legend of 6C is supplemented. The details are: Using the same RT-qPCR results as in Figure 6A, a histogram of representative genes were generated to analyze significance. *Brassica rapa* growth varied significantly under Cd stress, with different lowercase letters indicating significant differences (p<0.05). The *x* axis represents different concentrations of Cd stress treatment, and the *y* axis represents relative expression (C) (Line 793-797). All the legends are checked, and the legends of Figure 2B (Line 776-778) and Figure 7B are improved (Line 805-806).
(9) Whether the two graphs (Fig 6A and 6C) come from same RT-qPCR results? Please provide explanation.
Response to comment: Thank you very much for your professional advice. The two graphs (Fig 6A and 6C) come from same RT-qPCR results. The details are: Using the same RT-qPCR results as in Figure 6A, a histogram of representative genes were generated to analyze significance (Line 793-794).
(10) I suggest reorganizing the wording of the entire text, as some sentences are too long and confusing.

Response to comment: Thank you very much for your professional advice. According to the reviewer's suggestion, we reorganize the wording of the full text. We simplify the sentences that are too long and remove the repetitive content. It makes the full text easy to understand and easier to read.
